# “Highly committed but overwhelmed by constraints”: a qualitative study of officials’ and health staff’s perspectives on the prison mental health system in Cambodia

**DOI:** 10.1186/s12889-026-28046-0

**Published:** 2026-06-19

**Authors:** Puthy Pat, Kerstin Edin, Bhoomikumar Jegannathan, Miguel San Sebastian, Linda Richter Sundberg

**Affiliations:** 1 Department of Epidemiology and Global Health, Umeå University, Umeå, Sweden; 2 Centre for Child and Adolescent Mental Health (Caritas-CCAMH), Takhmau, Cambodia

**Keywords:** Mental health system, Prison, Cambodia

## Abstract

**Background:**

Prisoners have been identified in various international studies as one of the populations most vulnerable to mental health problems, yet the system’s response has been notably limited. Enhancing the prison healthcare system to effectively address the challenges involved a complex undertaking compounded by the prevailing constraints and stressors faced by both inmates and prison staff, particularly in low- and middle-income countries such as Cambodia, where research is lacking.

This study aimed to examine the perceptions of prison officials and healthcare staff regarding the health system’s challenges in addressing prisoners’ mental health and well-being and to explore strategies to improve the Cambodian prison healthcare system.

**Methods:**

Three focus group discussions were conducted in three prisons located in various regions of Cambodia with a total of 15 participants (11 prison health staff and four prison officials). A qualitative content analysis was applied to the collected data.

**Results:**

Despite limited resources, the current prison healthcare system managed to provide basic healthcare, refer severe cases, and implement certain preventive measures. However, our study revealed a lack of mental health focus within the system, although certain emotional support was provided by healthcare staff to prisoners with mental health issues. Staff displayed commitment but expressed a sense of resignation due to skills and resource constraints. Participants recommended various strategies, such as increasing awareness and offering vocational training to prisoners and room leaders, intense training for healthcare staff, and enhancing governance and external support to strengthen the prison healthcare system to foster the mental health and well-being of prisoners.

**Conclusions:**

Addressing the mental health needs within the prison setting in Cambodia demands an urgent reform of the current prison healthcare system. This entails a comprehensive collaborative effort among governmental institutions, placing emphasis on enhancing the skills and capabilities of inmates, room leaders and prison staff. Moreover, it is also essential to establish stronger partnerships with external organizations to improve health system governance.

## Background

Enhancing the prison healthcare system to effectively address and strengthen mental health and overall well-being within prison settings is a complex task given the high prevalence of mental health problems among prisoners and the continuous stressors they are exposed to [1, 2]. Prisoners in low- and middle-income countries (LMICs) are reported to have significantly more mental health problems than prisoners in high-income countries (HICs) [3] as well as the general population [4], and Cambodia is no exception [5]. Young prisoners in particular are found to be most vulnerable to both physical and mental health problems [6]. The response of the prison healthcare system is often limited [7], with services focused on curative rather than preventive activities and on physical rather than mental health [8]. A scoping review on mental health in prison settings during the COVID-19 pandemic found that limited and often unavailable mental health services had a significant adverse effect on the mental health and well-being of those who were incarcerated [9].

Research from HICs has shown that there are significant disparities in the quality of healthcare for the general population in comparison to in prisons where for example, inmates are more unlikely to receive treatment for mental illness [10]. A study from the UK shows that despite policy efforts, inequality in access to prison healthcare persists [10]. Similarly, studies from Australia indicate that the quality of prison healthcare varies to a large degree within the country, which has been linked to an under-resourced prison healthcare system [11]. In HICs, there is an attempt to bring reforms within the prison healthcare system by adding a new framework to assess performance and to promote informed decision-making for policy improvement and implementation that is relevant for low- and middle-income countries [2]. These frameworks may also provide valuable insights for adaptation in low- and middle-income countries (LMICs) [2].

In contrast, the prison healthcare system in LMICs faces even more challenges than that in HICs due to a lack of investment in infrastructure, health personnel, equipment, and medicines [4]. Health service provisions within prisons are often delivered through collaborations with external healthcare providers, such as local health centres and hospitals [4]. Establishing effective referral systems and ensuring a smooth transition of care between prison facilities and external healthcare institutions is therefore essential for maintaining the overall well-being of inmates [12].

The existing research on prison mental healthcare systems in these LMICs is limited. The few existing studies have reported a lack of mental health services. A qualitative study conducted in Bangladesh highlighted the absence of sufficient mental health services within the prisons, forcing the medical workforce to take on the responsibility of addressing mental health issues that they were not trained for [13]. Similarly, a study from Nigeria found that prisons lacked mental health services and that incarcerated individuals with mental health problems were referred to a federal mental health hospital for assessment and treatment [14]. In addition, research from Zimbabwe revealed that mental health services were lacking within prisons, even though overall healthcare services surpassed those available within the community in terms of both accessibility and quality of treatment [15].

Providing healthcare, including mental health services, within the prison environment presents a multifaceted challenge due to the demanding nature of the setting, which is marked by adverse conditions such as overcrowding [16], restricted personal space, violence, and curtailed freedoms [17]. Furthermore, constrained by limited resources, the prison healthcare system prioritizes physical over mental healthcare, resulting in inadequate training and staff proficiency to address mental health issues [2]. This situation, often complicated by the shortage of staff, heavy workloads, and the presence of psychosocial challenges, including issues such as compassion fatigue, work-related stress, and burnout among prison personnel, as reported from HICs, is also relevant for the LMIC context [18, 19]. All the above factors indicate the need for prison health system reforms to improve mental healthcare globally, particularly in LMICs.

Strengthening the prison health system is an essential public health component to better respond to the mental health and well-being needs of inmates [20]. It has been argued that improved prison healthcare services would not only lead to better health among prisoners but also to a long-term improvement in health outcomes in the general population at large [21]. Therefore, there is a need to understand the functioning of the system locally to identify its weakness and thus implement interventions to strengthen it [22]. However, as previously indicated, there is a lack of research and evidence in this area in LMICs, including Cambodia.

Research on prison settings in Cambodia is scarce. A study evaluating the effects of a life skills intervention on young prisoners showed no positive impacts in reducing mental health problems among this population [23]. In an effort to gain deeper insights into the mental health and well-being of young Cambodian prisoners, a qualitative study was conducted that highlighted a varied range of experiences, with some inmates reporting improvements in their overall health while others noted a deterioration [16]. The study pointed out that the mental health needs of prisoners often go unaddressed, and responses from the prison health system remain limited [16]. To improve the system and acquire a more comprehensive understanding of its current functioning, it is crucial to consider the perspectives of those responsible for health matters within the prisons.

The aim of this study was to examine how prison officials and healthcare workers perceive the challenges involved in addressing prisoners’ mental health and well-being and to explore potential strategies to enhance the Cambodian prison mental healthcare system.

## Method

### Study setting

In 2015, the low-income country Cambodia, reached the status of becoming a lower middle-income country [24]. The country, located in Southeast Asia, underwent the traumatic experience of more than three decades of civil war in which millions of people were killed [25]. The war destroyed the health system in the country, although it has been gradually restored since 1979 [26]. Overall, the health system still needs to be strengthened, particularly the prison health system.

There are 24 prisons spread all over the country [27], each with one health post commonly staffed with several type of personnel such as nurses, auxiliary nurses and/or health staff with very limited healthcare education and experience. The health posts provide basic healthcare for all prisoners and refer those with severe health problems to the nearest hospitals. In addition, non-governmental organizations (NGOs) collaborate with prisons to offer a variety of services to prisoners, such as health check-ups, the provision of medical supplies, health education, and legal aid. In addition, both the government and NGOs work together to provide vocational skills training for prisoners, such as hair cutting, carpentry, basic computer skills, and tailoring.

Overcrowding is one of the major concerns in Cambodian prisons [16], with approximately 50 prisoners accommodated in small rooms of approximately 50 square metres. Prisoners of varying ages, backgrounds, and criminal records are housed together within the same living quarters, with separate facilities designated for men and women. These prisons are not juvenile detention centres but rather prisons with mixed-age, accommodating both adult and young prisoners. Each room has a designated room leader, a prisoner who is assigned by the prison management team, to supervise the prisoners and performs various tasks – for example, distributing food, identifying health problems, reporting them, and referring coprisoners to the health post. Room leaders work under the supervision of prison officials, who are correctional staff responsible for the overall management, discipline, and security of the prison.

### Participants and procedure

Two-stage purposive sampling was employed to choose the participants. First, it was used to select the three prisons for this study located in Battambang, Kampong Cham, and Kandal provinces. Battambang lies in the northwest, Kampong Cham in the central lowlands of the Mekong River, and Kandal in the southeast near the capital, Phnom Penh where active mental health program in the communities and schools is being implemented. Despite their geographical differences, these regions share similar sociodemographic and economic profiles [28]. This approach aimed to capture variations in terms of prisoner context and, in that way, certain nuances within the Cambodian prison system. The central prison, located in the capital city of Phnom Penh that houses individuals convicted of the most serious crimes and political matters was not the focus of our study. Second, it was further utilized to identify and invite participants with health-specific responsibilities in the three selected prisons. The officials were invited to participate because of their relevant knowledge and insights into the prison health system; they had experience from being involved in prisoners’ health referrals and/or were responsible for providing health services.

Focus Group Discussions (FGDs) were chosen as the data collection method, allowing prison staff to share and explore both individual and collective experiences regarding physical and mental health in prison settings. Given the sensitivity of discussing mental health in a restricted setting, FGDs also offered greater confidentiality and reduced the risk of participants being identified compared to in-depth interviews. Three FGDs, one in each of the three selected prisons, were conducted. The FGDs included a mix of prison health staff and prison officials, with a total of 15 participants — between 4 and 6 individuals in each group. Participants varied in age, gender, professional roles, and years of experience. This diversity provided a range of perspectives on prison healthcare challenges and practices. Among them were 11 prison health staff members responsible for health-related services and 4 prison officials in charge of overall prison management and security. The mean age was 29.5 years while the mean year of experience was 12 years. This diversity provided a range of perspectives on prison healthcare challenges and practices. The FGDs were conducted from September to October 2021.

To plan for the FGDs, the researchers visited one of the three prisons and its health post to determine the overall prison health system situation, the health post environment, available medical resources, and equipment, and the accessible space for the FGD discussions. Pilot interviews with two prison health staff members were organized to assess the accuracy of the discussion guide and to gain insight into their perspectives concerning the mental health and well-being of the prisoners as a whole, including young prisoners. In this study, young prisoners refer to those aged between the age of 15 and 24 years as per definition of young people by World Health Organisation (WHO) [29]. The FGD guide included questions relating to young prisoners because this group is considered particularly vulnerable to mental health issues, such as stress, anxiety, and suicidal behaviour. This has been identified in prior research elsewhere [29] and in Cambodia [5]. The final semi-structured discussion guide was organized into the following sections: perceptions of the general health and mental health situation of the young prisoners; current responses of the prison health system to their health needs; the prioritized strategies and interventions needed, such as training opportunities; and the availability of and need for resources.

FGDs were held in either the prison library or health post to maximize the level of privacy and minimize potential disruptions. The prison management authorities and the participants permitted the audio recording of the FGDs. The discussions lasted approximately one hour for each group, and the conversations were conducted in Khmer, the native language of the participants, the moderator (first author) and the note-taker (research assistant).

### Analysis process

A qualitative content analysis using an inductive approach was employed to analyse the data inspired by the methodology formulated by Graneheim and Lundman [30]. The analysis utilized both manifest and latent content to gain a comprehensive understanding of the data. Initially, the analysis focused on the manifest content, in which the interpretation levels were close to the transcripts, particularly during the process of condensing meaning units and coding. However, the analysis also applied a latent approach at a later stage, which involved interpreting the underlying or implicit meaning of the data to finally and entirely generate the categories and themes.

The first author manually transcribed the audio recordings. The transcripts were translated into English from Khmer by a professional translator, while the first author cross-checked and verified the translations for accuracy. The first step of the analysis included reading the transcripts to enable the authors to familiarize themselves with the data. The second step involved the careful extraction of meaning units, i.e., words or parts of the transcript that had a shared meaning. In this step, meaning units were also labelled with codes. During this process, the first author coded the data directly from the original Khmer transcripts to avoid misunderstandings and ensure that nuanced meanings were accurately captured, while all the co-authors used the English version for consistency and clarity on development of condensed meaning units, sub-categories, categories and themes. In the third step, codes with similar directions and contents were first sorted into subcategories and then categories. In the final step, themes were developed by linking the categories and their meanings (Table 1). The coded data and emerging themes were reviewed and discussed by all authors to ensure consistency and analytical rigor. All authors contributed to refining the themes and interpreting the findings, thereby strengthening the credibility and validity of the analysis. The process of formulating themes included an interpretative process where the latent expressions and meanings from the codes and categories were linked and abstracted. A constant comparison and cross-check between the audio recordings, memos, and transcripts in both English and Khmer versions were performed by going back and forward throughout the whole process of data analysis to ensure that the content and meaning of the FGDs were accurately captured [31].

**Table 1:** Example of content analysis process

Condensed meaning units	Subcategories	Categories	Theme
…I was personally passionate about this job…	Full of compassion	Highly committed and experienced officials	*Compassion*,* commitment*,* and despair*
…I’ve been working in this prison almost 30 years, so a lot of experience…	Highly experienced staff		
…we never have enough medicines and equipment…	Inadequate medical supplies	A sense of despair due to challenges	
…I got a degree in law, but I work in the health post with a few days’ training on health…	Lack of capacity		

To ensure methodological rigor, the study adhered to the criteria of credibility, dependability, confirmability, and transferability as outlined by Graneheim and Lundman [30]. Reflexivity involves the researcher’s continuous reflection on how the researcher’s subjectivity and previous experiences shapes the research design, data collection, and interpretation. The researcher’s reflexive self-awareness and transparency is essential for enhancing the credibility and rigor of qualitative studies. The first author and two co-authors possess academic and clinical backgrounds in psychology, child and adolescent psychiatry, public health, and mental health research, with prior experience implementing psychosocial interventions within prison settings in Cambodia. The other two co-authors hold professorial positions in public health and health care. This combined expertise and familiarity with the local context facilitated the establishment of rapport with participants and enabled a deeper understanding of the prison dynamics.

## Results

Three themes emerged from the analysis of the FGDs: [1] Informal care and resource constraints shaping prison health system responses; [2] Compassion, commitment, and constrained capacity in prison healthcare; and [3] System-level strategies for strengthening prison mental healthcare under resource constraints.

### Theme 1: Informal care and resource constraints shaping prison health system responses

The first theme underscored the crucial role played by self-care practices, which involved the participation of prisoners’ families and the roles of room leaders in complementing the inadequacies of the prison healthcare system, particularly in dealing with mental health issues. These components were described as pivotal in fostering and sustaining the overall well-being and mental health of prisoners within the prison setting. However, it was noted that prison healthcare services did not solely focus on basic curative treatments; they also incorporated specific health promotion initiatives.

This theme highlights how limited formal healthcare resources within the prison system are supplemented by informal care practices and alternative support mechanisms. The findings showed that prisoners’ health and wellbeing, particularly mental health, were shaped by a combination of self-care, family support, and intermediary roles such as room leaders. Together, these elements formed a system of shared responsibility that compensated for gaps in formal healthcare provision. At the same time, the prison system incorporated basic health promotion activities, reflecting an attempt to balance constrained resources with preventive care.

### Self-care and family support as complements to formal healthcare

Participants described how prisoners frequently relied on self-care practices and family support as first-line responses to both physical and mental health concerns. Families played a central role by providing medicines and emotional support, often acting as the initial point of contact, particularly for younger prisoners. In parallel, prisoners engaged in coping strategies such as physical activity, social interaction, and recreational activities such as playing football, exercising, sharing jokes, and watching TV to manage stress and maintain wellbeing.

*Prisoners who have minor problems just call their family to get medicine for themselves*,* they do not come to us unless there is no improvement*,* like still having a fever or headache. […] (FGD 3)*.

When health concerns persisted, prisoners turned to room leaders, who acted as intermediaries between inmates and prison authorities. Room leaders identified health needs and facilitated access to formal care by notifying prison officials, who then coordinated referrals to healthcare staff. This layered pathway illustrates a system in which responsibility for health is distributed across prisoners, peers, families, and institutional actors.

*The room leaders identify those who have health problems and then inform the officials in charge in that building*,* and they make appointments with us. (FGD 1)*

### Limited formal healthcare and referral pathways

Formal healthcare services within the prison were primarily limited to basic primary care for common conditions. More severe or complex cases (such as seizures, unconsciousness, or cardiac problems) were referred to external hospitals, indicating reliance on external systems for advanced care.

*We may provide primary care for fevers and noncritical illnesses such as a cold*,* cough*,* pain on the right side of the stomach*,* etc. If we suspect any critical conditions*,* we will send the patient to the hospital […] (FGD 1)*.

Mental health services were underdeveloped and almost non-existent. When it came to mental health problems, such as severe stress, anxiety disorders and/or depression, the staff made efforts to counsel those prisoners by advising them to ignore unnecessary thoughts, encouraged them to practice behavioural coping strategies in exercise or read books and allowed them to communicate with their families by phone rather than offering specialised care. However, prisoners with severe mental disorders, such as psychosis, would be transferred to a hospital for appropriate treatment.

*For me*,* I tried to advise some prisoners with stress or anxiety or sleeping problems*,* but it is not easy. I need to keep confidentiality*,* and I told them not to think too much and just let it go. […] (FGD 3)*.

These findings suggest that while referral mechanisms exist, the scope of in-prison care, especially for mental health, is limited, reinforcing reliance on informal support systems described above.

### Health promotion within constrained systems

The participants indicated that the prison healthcare personnel typically conducted health education sessions for all inmates on a monthly or bimonthly basis as a preventive measure. This education primarily focused on healthy behaviours and lifestyles that could lead to positive health outcomes. It covered aspects such as personal hygiene, including cleaning rooms, toilets, and other shared spaces, as well as proper food-handling practices. To ensure compliance with these routines, prison officials regularly supervised the room leaders and prisoners in each room.

Despite resource limitations, health promotion activities formed an integral component of prison healthcare. Regular health education sessions focused on hygiene, healthy behaviours, and disease prevention, with room leaders and prison staff playing a role in reinforcing these practices. This reflects an institutional effort to maintain basic health standards through preventive strategies.

*We not only treat them*,* but we also educate them on health or healthy living*,* such as hygiene and good living habits. Then*,* we check whether they do it or not […] (FGD 3)*.

Additionally, prison staff proactively encouraged family support as a means to prevent and reduce health problems and/or improve mental health. This support involved facilitating phone calls, family visits to the prisons, and allowing the supply of food and other essential items by the relatives.

*When their family visits them*,* they feel happier*,* so we try to encourage their families to come more often. We do not have enough food or cleaning materials for them*,* so if they are from rich families*,* we also encourage their families to continue supplying them. (FGD 2)*

Facilitating visits, communication, and the provision of essential goods served both practical and psychosocial functions, helping to mitigate the effects of material shortages and emotional distress.

### Theme 2: Compassion, commitment, and constrained capacity in prison healthcare

This theme captures the tension between the strong personal commitment of prison staff and the structural constraints that shape their work. Participants described a deep sense of responsibility, compassion, and professional dedication to prisoner wellbeing. At the same time, persistent institutional and systemic challenges limited their ability to provide adequate care, contributing in some cases to frustration and a sense of resignation. These dynamics were particularly evident in relation to the care of young prisoners, for whom participants expressed heightened concern.

### Commitment, experience, and compassion as drivers of care

Participants consistently portrayed themselves as highly committed and experienced professionals, many with over a decade of service. Their accounts emphasised a strong sense of duty to both the nation and the incarcerated population, with compassion seen as essential to fulfilling their roles effectively. This moral and professional commitment appeared to underpin their continued efforts to provide care despite challenging working conditions.

*I am personally passionate about this job. I want to serve my nation and people in the prison. If we do not have compassion to do so*,* we should resign. (FGD 1)*

### Institutional and systemic challenges

Many challenges, both from inside and outside the prisons, were expressed by the participants. One important challenge linked to characteristics within the prisons, was the limited resources and training opportunities. They complained about the scarcity of staff, resources, and limited competency in providing healthcare due to a lack of training. A lot of variation in clinical education among the prison health staff was described, with some being nurses while others had no healthcare training at all in their background. In addition, there was a lack of opportunities for health staff to engage in clinical case discussions, where they could collaborate and gain new knowledge and insight to further improve the quality of care and treatment for sick prisoners. Additionally, from the participants, it appeared that there was neither a continuous capacity-building programme nor an available plan for updating training opportunities.

*We do not have enough medicines and other equipment to perform health check-ups for our prisoners. (FGD 2)*

*We used to receive some training on HIV/AIDS and TB many years ago but no training at all on mental health. We also used to have training on illicit drugs for a few days. (FGD 1)*

In term of external challenges, participants emphasized issues related to collaboration and support from external agencies. Collaboration with the outside healthcare system was also described as demanding. The waiting times at the hospitals were too long, and the officials were concerned about security issues related to the prisoners when visiting external health facilities. Additionally, the prison health staff had to request medicines monthly from the closest health centre, but the medicine supplies never corresponded to their needs; therefore, the prison health posts were always faced with a shortage of medicines and medical equipment.

*[…] it is difficult to collaborate with the hospital*,* and I have tried to explain to them that we don’t have enough staff for security*,* but we still have to wait for a very long time […] the health centre kept complaining about us*,* saying that our reports were wrong and always late*,* they informed their boss and blamed us […] they didn’t look at our reports and requests*,* so we never get the right medicines or enough medicines. (FGD 3)*

### Emerging sense of constraint and resignation

Despite their strong commitment, participants also expressed feelings of frustration and, at times, resignation. These sentiments were linked not only to resource and system constraints but also to perceived challenges in engaging prisoners themselves. Some participants described prisoners as resistant to advice or behavioural change, which contributed to a sense of limited impact and uncertainty about how to improve health outcomes within the prison setting.

*[…] They (prisoners) never listened to their parents*,* so how could they listen to me? There are laws out there*,* but they still committed an offence. When they were gathered here*,* they would not listen to us*,* so I don’t know how to improve their well-being […] (FGD 1)*.

### Theme 3: System-level strategies for strengthening prison mental healthcare under resource constraints

This theme reflects participants’ perspectives on how prison health systems could be strengthened to better address mental health and overall wellbeing, while recognising persistent resource limitations. Their accounts point to a multifaceted approach that combines awareness-raising, capacity building, structural improvements, and enhanced coordination across system levels. These proposed strategies highlight both immediate, pragmatic solutions and longer-term systemic reforms.

### Raising awareness and promoting skills for mental health and wellbeing

Participants emphasised the need to strengthen mental health awareness among prisoners, particularly younger individuals, as a foundational step. This included increasing understanding of stress, emotional distress, and coping strategies. Beyond awareness, they highlighted the importance of structured educational programmes focused on self-regulation, emotional management, and life skills development.

*I think to improve their (prisoners’) mental health*,* we need to have a programme to raise their understanding of mental health*,* emotions and thoughts […] (FGD 3)*.

Furthermore, participants viewed the provision of more comprehensive education on health and mental health as a crucial second step toward improving prisoners’ overall well-being. They emphasized the need for structured programmes and instructional courses that foster self-awareness, self-regulation, emotional management, anger control, and a more constructive mindset, alongside a curriculum focused on essential life skills.

*I think it is important to make them aware and train their minds*,* control themselves*,* and regulate their emotions because it is difficult to live here and they are bored*,* confused and have uncertainty about their future […] (FGD 3)*.

Vocational training was also identified as a key component of mental wellbeing. Participants viewed skills development—such as technical or trade-based training—not only as preparation for reintegration after release but also as a way to reduce stress, uncertainty, and negative thinking during incarceration. Together, these approaches reflect an emphasis on prevention and empowerment within constrained settings.

*Prisoners regret their past and have negative thinking about their future*,* so all of them have mental health problems. We have to provide vocational training skills for them like handicraft*,* so they feel happy*,* so that they have no mental health problems […] (FGD 3)*.

### Addressing structural and environmental determinants of health

Participants underscored the importance of improving basic living conditions within prisons, including space, hygiene, and access to adequate food and water. These structural factors were seen as directly influencing both physical and mental health.

*To help improve health conditions*,* enough food must be provided*,* and the cells should be expanded. The environment must be gradually improved […] (FGD 1)*.

Recreational activities including a wide range of options, from outdoor exercises such as sports to indoor pastimes such as watching television were also highlighted as important for emotional and social wellbeing. Opportunities for physical exercise, social interaction, and leisure were perceived as helping prisoners cope with distress and adapt to prison life. These findings point to the role of environmental and social conditions as integral components of mental healthcare.

*They play football*,* watch TV*,* help each other*,* and exercise together. Therefore*,* they feel good about living here and become well-adjusted with others […] (FGD 2)*.

### Need for strengthening skills building for officials and room leaders

A recurring theme was the need to strengthen the competencies of prison staff in areas related to mental health, including counselling, psychoeducation, and basic clinical management. Participants expressed a lack of confidence in responding to mental health needs and prioritised training as a critical gap, sometimes even over material resources.

*Many of our staff also face mental health challenges*,* and when we encounter prisoners with similar problems*,* we often don’t know how to respond. We need more training — especially on how to dispense medicines — rather than only being provided with the medicines. (FGD 3)*

Room leaders were identified as key actors within the prison system, serving as the first point of contact for many prisoners. Strengthening their capacity through training was seen as an efficient strategy to extend the reach of health interventions. Participants proposed cascading training models, where trained individuals disseminate knowledge to others, as a practical solution within resource-constrained environments.

*There are approximately 2*,*000 prisoners*,* so we cannot provide health education sessions to all of them. We can invite room leaders for health education programmes*,* and they further educate 10 other prisoners*,* and these 10 prisoners further educate 10 other prisoners or 20 or 30 other prisoners. (FGD 1)*

### Promoting leadership and management for enhanced prison health

Participants highlighted the need for clearer roles and responsibilities within the prison health system, as well as increased staffing and improved management structures. Importantly, they emphasised that strengthening prison healthcare should not rest solely on prison staff but requires coordinated support from multiple sectors, including relevant ministries, such as the Ministry of Interior, the Ministry of Health, and the Ministry of Justice.

Collaboration with external health services was also identified as essential. Participants called for improved coordination mechanisms, such as regular meetings and clearer communication channels, to address challenges related to referrals, supplies, and service delivery. In addition, partnerships with non-governmental organisations were viewed as critical for supplementing limited resources, providing technical expertise, and supporting capacity building.

*For me*,* it is not only our work to improve health systems here but also the shared task with the ministry level*,* and if there is no intervention from them*,* we cannot fulfil our needs to strengthen the healthcare system. We need financial and technical support from them. (FGD 3)*

### Family support as a core component of care

Family support was consistently described as central to prisoners’ mental wellbeing. Participants emphasised the importance of facilitating communication, visits, and material support from families. At the same time, they recognised the need for additional institutional support for prisoners without family networks. This highlights the role of social support systems—both formal and informal—as integral to prison healthcare.

*[…] We advise them to talk to their families. They are allowed to take 30 min break per day […] we want to permit the family members to meet and give food to the prisoners as it will help improve their health conditions. (FGD1)*

### Strengthening collaboration across levels of the health system

Participants emphasised the importance of improving coordination between prison health services and the broader healthcare system. Prison health posts rely on external health centres for reporting, referrals, and the provision of medicines, making effective collaboration essential for service delivery. However, participants described gaps in communication and coordination, which limited the responsiveness of external support.

To address these challenges, they proposed strengthening collaboration through clearer understanding of roles and responsibilities across system levels, as well as establishing regular coordination mechanisms. Suggested strategies included joint meetings involving prison health posts, district health offices, health centres, provincial health authorities, and other stakeholders to collectively identify challenges and develop solutions.

*All service providers should cooperate with each other and have a meeting to discuss some issues. Prison health posts*,* the health district offices*,* health centres*,* provincial health departments and NGOs like you should meet to find some solutions […] (FGD 3)*.

In addition, partnerships with non-governmental organisations (NGOs) were viewed as critical in addressing resource and capacity gaps. Participants highlighted the role of NGOs in providing medical supplies, technical expertise, and training, particularly in areas such as mental health where internal capacity is limited. These collaborations were seen as essential complements to formal health system support, helping to strengthen service provision in resource-constrained settings.

*[…] if there are NGOs to come and help*,* that is good. We don’t have skills for mental health*,* and it is necessary. We lack this resource*,* so NGOs could help us. (FGD 2)*

## Discussion

This study explored the perceptions of prison officials and healthcare workers regarding the challenges of addressing prisoners’ mental health and well-being, as well as potential strategies to improve prison mental healthcare in Cambodia. The findings revealed that mental health services were underdeveloped and virtually absent within the prison healthcare system. However, certain healthcare personnel did make efforts to provide support to prisoners with mental health problems. When becoming unwell physically and psychologically, prisoners often prioritized self-care and sought support from their families before turning to prison healthcare services, often facilitated by the room leaders. Despite facing a range of challenges, the current Cambodian prison healthcare system manages to provide basic health services, offer referrals for severe health conditions, and implement some preventive measures. These challenges appeared to foster a sense of dedication among the staff, albeit tinged with a degree of resignation towards the prevailing health system challenges within the prisons. Our research also revealed that participants had limited training opportunities and a low educational background in health-related subjects. Their recommendations to reform the prison healthcare system to better respond to mental health and well-being in the prisons seemed ambitious in the context of resource constraints but they were closely aligned with numerous international studies and evaluations. The suggested measures encompassed providing raised mental and health awareness and vocational training to prisoners and room leaders and capacity building for prison health staff, with a focus on strengthening the health workforce within the system. Furthermore, there was a strong emphasis on the need to improve the governance of the prison healthcare system and to seek external support for its enhancement. These findings contribute to a broader understanding of how, in resource-constrained settings, both inmates and staff adapt to fill service gaps through personal resilience and social support systems.

### Self-care and family support complement prison healthcare

Our research found that incarcerated individuals resorted to self-care and relied primarily on family support to tackle their physical and mental health concerns. A qualitative study conducted in the United Kingdom discovered similar findings: prisoners employed effective self-care techniques, such as forming personal and group connections within the prison environment, as their primary approach to deal with physical and mental challenges during times of illness and distress [32]. Furthermore, participating in sports activities, which can be seen as a self-care practice, has been identified as having a positive impact on enhancing resilience, mental well-being, and overall health [33]. Another study that investigated health and well-being strategies among offenders in England also highlighted that individual prisoners adopted daily routines, another form of self-care, as a way to divert their attention and temporarily disengage from their psychological challenges before seeking professional help [34]. Family visits seemed to complement self-care strategies within the prison setting and reduced mental health challenges among the prisoners [35]. A study from Iran highlighted the relationship between family cohesion and psychological well-being among male prisoners [36]. These findings highlight the potential of integrating family involvement and structured self-care initiatives (e.g., sports, peer groups, and health education) into prison health programs.

### Compassion, commitment, and despair due to prison healthcare system challenges

Persistent gaps in mental health service provision, largely due to the limited capacity of prison staff and poor resource allocation were identified. Although some staff members provided basic counselling or referrals, formal mental health programs were minimal. These findings mirror patterns seen in LMICs, including Bangladesh [13], Zimbabwe [15], and Nigeria [14].

Two primary challenges within the prison health system – the shortage of resources and the difficulties in collaborating with other agencies such as hospitals or health centres – were identified. These findings align with numerous international studies. For instance, studies carried out in the United Kingdom prisons highlighted resource shortages, including insufficient staff training and funding [7, 37]. Another study focused on mapping mental health services within Australian prisons proposed that the prison mental health system should receive sufficient resources, effectively address the intricate requirements within the prison environment, and guarantee the uninterrupted provision of healthcare as individuals reintegrate into the community [11]. The situation in LMICs is equally challenging. A qualitative investigation conducted in Zimbabwean prisons highlighted issues related to accessibility and connectivity with outside hospitals [15]. However, the provision of increased resources and continuing healthcare until reintegration into the community can be challenging, particularly in countries with limited resources, such as Cambodia. These findings highlighted the importance of developing practical, context-specific solutions rather than relying on imported models of care.

The underdevelopment of mental health services within Cambodian prisons reflects the broader systemic challenges of the national mental health system. Limited resources, a shortage of trained professionals, and gaps in infrastructure affect both incarcerated and non-incarcerated populations. Although some community-based mental health services exist [38], their accessibility and quality vary considerably. Nationally, Cambodia has only about 60 psychiatrists, equivalent to one psychiatrist per 260,000 people [39] and none of these professionals, nor other mental health specialists, are available within the prison system. Mental services are also provided through a few specialized centres, such as the Centre for Child and Adolescent Mental Health (CCAMH) and Transcultural Psychosocial Organization (TPO) Cambodia, which play vital roles in both service delivery and workforce training but there is a service need versus availability gap. This disparity underscores the need for national strategies to strengthen the access and quality service across all settings including the prisons. Situating prison mental health within this broader context provides critical insight into systemic barriers and the necessity for integrated program that address mental health needs in both community and correctional settings.

#### Enhancing prison mental healthcare

Participants emphasized the need for reform in several areas, including improving the physical environment, enhancing staff capacity, and strengthening system governance. Similar to our findings, studies from India and northwest England have shown that environmental interventions such as gardening can significantly enhance mental health and overall well-being in prison settings [40, 41]. Another study investigating the factors influencing health and healthcare accessibility in Zambian prisons underscored the necessity of enhancing physical structural conditions (less crowding and more sleeping spaces), as these elements were found to be linked with the physical and mental well-being of both incarcerated individuals and staff members [42].

In our study, participants provided a detailed description of the skills and capacity building needed in the prison setting. A scoping review focused on interventions aimed at improving prison healthcare similarly highlighted the positive effects of health education for both prison staff and inmates in bolstering the overall healthcare system [43]. Furthermore, a study addressing the needs and barriers within Canadian prisons proposed that training opportunities and programmes for correctional staff could contribute to mitigating mental health challenges among both staff and inmates [22]. However, while certain studies have identified positive effects of training and educational interventions on prison healthcare, there were also instances where the outcomes have been more varied and mixed. A systematic review of 19 studies examining 14 different intervention programmes aiming to improve mental health outcomes within prison settings showed that training interventions did not yield statistically significant improvements in the treatment and health services provided within the prisons [44]. The effectiveness of these interventions appeared to be contingent on a range of factors, including the nature of the training, the contextual conditions of implementation, and the distinct requirements and obstacles faced by the prison healthcare system [45]. All of this suggests that future interventions must be contextually adapted and evaluated systematically. Additional research is imperative to determine the optimal strategies for enhancing the skills and capacity of both prison staff and inmates, with the ultimate goal of improving prison mental healthcare.

Another recommended intervention identified in our study to enhance mental health and well-being within the prisons was the provision of life skills education for prisoners. A study from Portugal highlighted the benefits of life skills education for prisoners, particularly for juveniles, as it provided inmates with the tools and knowledge necessary to cope with the challenges of life in prison, improve their mental health, and increase their chances of successful reintegration into society after release [33]. However, our own research has shown no effect of this programme in reducing the mental health problems of young prisoners [23]. These contradictory results highlight the importance of carefully designing, adapting, and implementing life skills programmes to the specific needs and circumstances of the target population, taking into account factors such as age, gender, culture, and environmental factors. Evaluations of such programmes should also be rigorous and comprehensive to assess their impact and make necessary adjustments for maximum effectiveness. Further research in different contexts can help identify best practices and approaches for tailoring and implementing life skills programmes effectively in prisons,

Our current study found that a key component suggested by the participants for improving the prison health system was strengthening the governance of prison health, which involves collaboration among different ministries involved in prison functioning (such as the Ministry of Interior, Ministry of Health and Ministry of Justice). This finding is aligned with recommendations from the WHO and the United Nations Office on Drugs and Crime (UNODC), which have called for a whole-of-government responsibility and involvement from relevant agencies and ministries in prison health management and coordination [46]. Furthermore, a study on global prison healthcare governance found that effective governance and accountability for prison healthcare services are essential to address health disparities and provide qualified care in custody [47]. These findings highlighted the importance of a collaborative, coordinated, and accountable approach to prison health management to ensure the provision of adequate mental healthcare services for prisoners.

While the proposed strategies, including capacity building of prison staff, peer interventions, and inter-ministerial collaboration, are crucial, their successful implementation depends heavily on political commitment and the prioritization of mental health within national health policies. In Cambodia, the Mental Health Strategic Plan 2023–2032 has been developed, reflecting a growing recognition of the need to strengthen mental health systems. However, sustained political engagement and effective cross-ministerial coordination—particularly among the Ministries of Health, Interior, and Justice—remain essential to ensure long-term reforms and adequate resourcing for prison mental healthcare.

#### Methodological considerations

The trustworthiness of this study was bolstered through several precautions. To strengthen the trust between participants and researchers, the researchers engaged in informal discussions with the involved officials and healthcare staff prior to conducting the FGDs. During the FGDs, a strong emphasis was placed on ensuring voluntariness and safeguarding confidentiality. These measures likely contributed to encouraging participants to openly share their genuine perspectives, thus enhancing the study’s trustworthiness; however, involving mixed hierarchies within FGDs could curtail participants’ freedom of expression, and certain crucial issues may not have been raised. This aspect might impact the overall credibility of the study by potentially limiting the range of viewpoints and concerns brought to light.

In terms of ensuring dependability, we implemented measures to uphold a high level of documentation by meticulously recording detailed audio recordings and memos verbatim throughout both the data collection and analysis phases. Furthermore, the analysis process adhered to widely accepted qualitative content analysis standards [30], contributing to strengthening the reliability and consistency of the findings.

A comprehensive and transparent depiction of every phase of the research, from inception to the finalization of findings, may aid readers in comprehending the research process and potentially applying the insights to their own contexts. Additionally, for the purpose of capturing variations in prison conditions and prisoner situations, we purposefully selected three prisons representing diverse geographical regions within the country. Through this selection, the participating prisons were intended to serve as representatives of the broader prison landscape within Cambodia, contributing to increased transferability of the findings. However, it is important to note that the exclusion of the central prison, which houses individuals convicted of the most severe crimes and political matters, could restrict the applicability of the study’s findings to that specific setting. In addition, this study involved 15 participants and most of them had limited or no formal training in mental health. This lack of professional background may have influenced their understanding and interpretation of mental health issues among prisoners.

## Conclusion

The mental health needs of young prisoners are unmet in the existing prison healthcare system, and the various perspectives of prison staff concerning the overall health conditions in Cambodian prisons underscored the pressing need for health system reform to address the complex and diverse needs of incarcerated individuals, particularly their mental health. To bolster the prison healthcare system, it may be essential to implement a comprehensive capacity building programme, alongside establishing a collaborative partnership between governmental institutions and NGOs, with the aim of enhancing mental health and overall well-being within the prisons.

This collaborative effort should further concentrate on enhancing the skills and capabilities of all relevant parties, such as prisoners, room leaders, and prison staff. Additionally, fostering stronger partnerships with other stakeholders, such as healthcare centres and hospitals, becomes crucial to adequately meet the demands of prison health posts and provide improved medical care. Continued family support and the promotion of self-care strategies can also play pivotal roles in ameliorating prisoners’ mental health and overall well-being. Future research in Cambodia and other LMICs should explore feasible, contextually and culturally appropriate models of mental healthcare within prison settings. The focus should be on low-cost, scalable interventions as well as on examining and counteracting barriers to service delivery.

In summary, the enhancement of health systems within Cambodian prisons to better address the mental health and well-being of detainees necessitates a holistic and cooperative approach that involves the collective efforts of all stakeholders.

## Data Availability

The datasets used in the current study are not publicly accessible due to privacy and confidentiality concerns. However, they can be obtained from the corresponding author upon reasonable request.

## Abbreviations

FGDs: Focus group discussions
GDP: General Department of Prisons
HICs: High-income countries
LMICs: Low- and middle-income countries
NECHR: National Ethics Committee for Health Research
NGOs: Nongovernmental organizations
RGC: Royal Government of Cambodia
UNODC: United Nations Office on Drugs and Crime
WHO: World Health Organization
